# Global and regional burden of disease and injury in 2016 arising from occupational exposures: a systematic analysis for the Global Burden of Disease Study 2016

**DOI:** 10.1136/oemed-2019-106008

**Published:** 2020-02-13

**Authors:** GBD 2016 Occupational Risk Factors Collaborators, Tim Driscoll

**Keywords:** workplace, cancer, noise, ergonomic, respiratory tract diseases

## Abstract

**Objectives:**

This study provides an overview of the influence of occupational risk factors on the global burden of disease as estimated by the occupational component of the Global Burden of Disease (GBD) 2016 study.

**Methods:**

The GBD 2016 study estimated the burden in terms of deaths and disability-adjusted life years (DALYs) arising from the effects of occupational risk factors (carcinogens; asthmagens; particulate matter, gases and fumes (PMGF); secondhand smoke (SHS); noise; ergonomic risk factors for low back pain; risk factors for injury). A population attributable fraction (PAF) approach was used for most risk factors.

**Results:**

In 2016, globally, an estimated 1.53 (95% uncertainty interval 1.39–1.68) million deaths and 76.1 (66.3–86.3) million DALYs were attributable to the included occupational risk factors, accounting for 2.8% of deaths and 3.2% of DALYs from all causes. Most deaths were attributable to PMGF, carcinogens (particularly asbestos), injury risk factors and SHS. Most DALYs were attributable to injury risk factors and ergonomic exposures. Men and persons 55 years or older were most affected. PAFs ranged from 26.8% for low back pain from ergonomic risk factors and 19.6% for hearing loss from noise to 3.4% for carcinogens. DALYs per capita were highest in Oceania, Southeast Asia and Central sub-Saharan Africa. On a per capita basis, between 1990 and 2016 there was an overall decrease of about 31% in deaths and 25% in DALYs.

**Conclusions:**

Occupational exposures continue to cause an important health burden worldwide, justifying the need for ongoing prevention and control initiatives.

**Key messages**

**What is already known about this subject?**

- Occupational risk factors have previously been identified as being an important cause of disease and injury burden.
- However, there has been no comprehensive update since 2000 using burden of disease methodology.

**What are the new findings?**

- Carcinogens; particulate matter, gases and fumes; and risk factors for injury were responsible for the greatest burden in terms of deaths.
- Ergonomic exposures associated with low back pain, injury risk factors and noise were responsible for the greatest overall burden (as measured by disability-adjusted life years (DALYs)).
- Men had much higher rates of deaths and DALYs than women.
- There was an overall reduction in rates of deaths and DALYS between 1990 and 2016.

**How might this impact on policy or clinical practice in the foreseeable future?**

- Occupational exposures are largely controllable and should be a focus for prevention activity in all regions.

## Introduction

A safe and healthy work environment is a fundamental right of all workers. Burden of disease studies are an important source of information on the level of ill health in communities and on the risk factors that contribute to this ill health.^1^ The results provide guidance to policymakers in terms of where resources might best be used and can provide insight into the effectiveness or otherwise of past interventions. The results can also be used to underpin studies of the cost of injury and illness.

Estimates of the worldwide burden of occupational disorders (diseases and injuries) suggest occupational risk factors make an important contribution to the burden of ill health,^2 3^ and there are large variations in and between countries in the estimated incidence of occupational disorders.^4^ Global estimates of economic costs arising from occupational disorders vary between 1.8% and 6.0% of gross domestic product.^5^ In principle, most occupational disorders can be prevented by means of control measures targeted at relevant risk factors.

Prior Global Burden of Disease (GBD) studies have estimated the global burden for 1990^6^ and 2010,^7^ with the 2010 GBD study updated several times.^8–11^ Occupational risk factors formed one group of risk factors that was included in the GBD-related Comparative Risk Assessment (CRA) study, focused on 2000.^3^ The occupational risk factors included in GBD 2016 were carcinogens; asthmagens; particulate matter, gases and fumes (PMGF); secondhand smoke (SHS); noise; ergonomic risk factors for low back pain; and risk factors for occupational injury. The study attempted to overcome some of the shortcomings associated with the earlier analyses, including some additional risk factors and associated outcomes and using modified methodology. The main GBD risk factors paper provides some high-level information on occupational risk factors.^10^ This paper and two companion papers^12 13^ describe in more detail the methods, results and strengths and limitations of the occupational risk factor analysis from GBD 2016.

## Methods

### General approach

The GBD 2016 study evaluated the burden of disease (deaths or disability-adjusted life years (DALYs)) attributable to past exposure to various risk factors. Attributable burden was estimated by comparing observed health outcomes to those that would have been observed if a counterfactual level of exposure (the theoretical minimum risk exposure level (TMREL)) had occurred. ‘Occupational exposure’ was defined as being experienced in the course of an activity undertaken for pay, profit or kind. It excluded home duties. The burden of occupational disease for each risk factor–outcome pair was estimated using the population attributable fraction (PAF), that is, the proportion of deaths or DALYs that would not have occurred if exposure was at the TMREL; this was then used to estimate attributable numbers of deaths or DALYs. The PAF requires information on the relative risk of the disease due to the exposure of interest and the proportion of the target population exposed. The risk estimates (relative risks or ORs) were primarily obtained from published meta-analyses or pooled studies or, where these did not exist, key single studies were used. Where single studies were used, the chosen study was the best quality study (as judged by the Occupational Risk Factors Expert Working Group) with exposure circumstances that were assessed as most closely matching those assumed in the GBD study. For carcinogens, PMGF, noise and ergonomic risk factors related to low back pain, most relative risks used in the analysis compared occupationally exposed to non-occupationally exposed, assuming similar exposure durations and intensities across countries and regions. The same relative risks were assumed to apply for calculation of burden of deaths and of DALYs. High-income countries were defined as countries in the Australasia, high-income North America, Western Europe, and high-income Asia Pacific regions, and low and middle-income (LMI) countries as all other countries. Risk factor–outcome pairs were selected where there was judged to be reliable data on both the exposure and the associated risk measures, the level of evidence supporting a causal association was sufficiently strong and the resulting burden was likely to be more than trivial.

PAFs were estimated for each age-sex-country group using the equation based on Levin^14^:

$$PAF=\frac{\sum_{x=1}^{n}RR\left(x\right)P\left(x\right)-1}{\sum_{x=1}^{n}RR\left(x\right)P\left(x\right)}$$

where *P(x*) is the proportion of persons exposed at level x in the relevant population and *RR*(*x*) is the relative risk corresponding to exposure level x.

Relative risks based on high exposed and low exposed, relative to those with background exposure, were used in lieu of more sophisticated measures of risk because the necessary quantitative information on exposure was not available. The age-sex-country-specific PAFs were multiplied by the total number of deaths in 2016 in the relevant age-sex-country group to produce the number of deaths in the age-sex-country-specific group for the relevant risk factor–outcome pair. These deaths in specific groups were summed to produce the total number of deaths from a given outcome resulting from the relevant exposure. This total was divided by the total number of deaths from all causes for a given outcome to produce an all-age PAF for the relevant risk factor–outcome pair. This was done separately by sex and for both sexes combined, and separately by country and region and for all regions combined. PAFs based on DALYs were calculated in the same way. A combined PAF for an outcome with multiple contributing exposures was calculated using the standard product-sum approach.^14 15^

$$PAF_{combined}=1-\Pi_{k}(1-PAF_{k})\;(for\;k\;exposures).$$

A different approach was used for occupational injury risk factors and resultant injuries, with information on the number of injuries coming directly from International Labour Organization (ILO) information, and for pneumoconiotic dusts and pneumoconiosis, with information estimated as part of the overall GBD estimates of prevalence and deaths for each included cause.

Results were calculated for all years from 1990 to 2016, inclusive; the 2016 findings are the focus of this paper. Only attributable burden in persons 15 years and above is included. Region-specific, sociodemographic index (SDI)-specific and global results are reported here. The SDI is a composite indicator of development status based on total fertility rate, mean education for those aged 15 years and older and lag distributed income per capita.^10^ The PAFs presented were based on DALYs unless otherwise indicated and were based on all ages. Per capita rates (age and sex standardised) were based only on persons aged 15 years and above, unless otherwise stated. The number, rate and proportion of the various outcomes each provide insight into different aspects of the burden, with the number reflecting absolute burden and the rates more useful when comparing burden between countries or between different time periods.

### Employment data

For carcinogens, PMGF, SHS and noise, the proportion of ever-exposed persons was based on the estimated proportion of each national population in the workforce, the proportion of the workforce in specific industries and the proportion of workers estimated to be exposed in that industry. For asthmagens and ergonomic risk factors, the proportion currently exposed was based on the proportion of the workforce in specific occupations rather than industries. Information on industry (nine categories), occupation (eight categories) and the proportion of the population which was working (the Economically Active Population (EAP)) for each country was obtained from the ILO Labour Force.^16^ Employment data from the year being examined were used for all risk factors except carcinogens, for which information on employment in previous years was also included to take account of latency and persistent risk. Available occupation and industry information was available by sex but not age. The EAP was available separately for males and females and for each GBD age group. Where the ILO data did not provide sufficient information for a country, information was obtained from subnational data sources, supplemented where necessary by modelling on a preselected list of covariates (such as log lagged distributed income, education per capita and urbanicity). In particular, for China, subnational data were extracted at the province level using census and national population sample surveys. For India, there were no national labour force data, resulting in the estimates being based on models driven by country-level covariates.

### Estimation of uncertainty

Ninety-five per cent uncertainty intervals (95% UI) were used rather than CIs. These attempt to take into account uncertainty from all input data components (exposure, relative risk, TMREL and burden), rather than just being based on random sampling, and were calculated as the 2.5th and 97.5th percentiles of 1000 draws taken from an assumed distribution of all relevant variables.^10^ Uncertainty intervals are primarily presented in detail in the tables to assist with the flow of the text.

More details on the methods used are provided in the online supplementary material, in companion papers on specific risk factors^12 13^ and on the GBD website, including those for specific countries, using the GBD Compare data visualisation.^17^

10.1136/oemed-2019-106008.supp1 Supplementary data

## Results

In 2016, there were an estimated 1.53 (95% UI 1.39–1.68) million deaths attributable to exposure to work-related risk factors included in this study. This represented 2.7% of all deaths in 2016 (3.1% of those aged 15 years or more). The risk factors resulting in the most deaths were PMGF and SHS resulting in chronic obstructive pulmonary disease (COPD) (30%), with carcinogens (23%), injury risk factors (22%) and SHS resulting in other diseases (22%) causing a similar number of deaths (table 1). The majority (77%) of the deaths occurred in males.

**Table 1:** Global occupation-attributable deaths, DALYs and PAFs by risk factor and sex, 2016 (number, per cent and proportion (95% uncertainty interval))

		Deaths				DALYs				PAFs*	
	**Males†**	**Females**	**Total**	**%‡**	**Males**	**Females**	**Total**	**%§**	**Males**	**Females**	**Total**
Carcinogens	274 175	74 566	348 741	22.8	5 560 418	1 639 432	7 199 850	9.5	4.5	1.8	3.4
	(215 339–333 928)	(60 870–89 775)	(282 253–414 071)		(4 375 804–6 734 491)	(1 312 494–2 017 294)	(5 813 091–8 641 244)		(3.6–5.5)	(1.5–2.2)	(2.7–4.0)
PMGF+SHS¶	343 122	116 958	460 080	30.0	7 969 986	2 717 967	10 687 953	14.0	21.1	10.6	16.9
	(270 900–422 000)	(87 100–153 300)	(381 500–551 300)		(6 518 000–9 469 200)	(2 143 400–3 328 600)	(9 019 900–12 517 000)		(17.4–24.8)	(8.5–12.9)	(14.3–19.7)
Injury risk factors	301 043	31 508	332 550	21.7	18 799 904	2 632 725	21 432 629	28.2	10.6	3.4	8.4
	(289 315–313 036)	(29 829–33 125)	(320 989–344 650)		(17 168 108–20 722 961)	(2 241 086–3 117 772)	(19 461 136–23 891 462)		(10.2–11.1)	(3.1–3.7)	(8.1–8.7)
Asthmagens	26 103	11 471	37 574	2.5	1 468 347	871 133	2 339 480	3.1	12.9	7.1	9.9
	(17 900–35 300)	(8700–15 200)	(28 400–47 900)		(1 141 200–1 874 300)	(666 100–1 109 600)	(1 860 900–2 923 300)		(11.5–14.3)	(6.3–8.0)	(9.0–10.7)
Pneumoconioses	18 997	2491	21 488	1.4	518 917	58 060	576 977	0.8	1.0	1.0	1.0
	(15 500–22 700)	(2100–3200)	(17 900–25 400)		(439 900–611 300)	(49 400–70 700)	(493 600–673 500)				
SHS (excluding cancer and COPD**)	222 933	109 065	331 998	21.7	7 622 593	3 685 843	11 308 436	14.9	–	–	–
	(147 461–304 921)	(68 890–152 887)	(216 952–455 510)		(4 850 498–10 571 425)	(2 202 327–5 270 793)	(7 041 695–15 788 968)				
Ergonomic risk factors	0	0	0	–	8 463 913	7 016 018	15 479 932	20.3	32.5	22.2	26.8
					(5 874 618–11 346 133)	(4 844 189–9 423 787)	(10 733 369–20 772 446)		(30.4–34.5)	(20.5–24.1)	(25.0–28.8)
Noise	0	0	0	–	4 711 557	2 396 720	7 108 277	9.3	25.1	13.7	19.6
					(3 294 182–6 488 167)	(1 680 298–3 296 175)	(4 978 557–9 802 692)		(23.2–26.9)	(12.8–14.8)	(18.2–21.1)
Total	1 186 372	346 058	1 532 431	100.0	55 115 652	21 017 906	76 133 558	100.0	4.2	1.9	3.2
	(1 066 502–1 311 010)	(299 889–398 048)	(1 387 905–1 684 933)		(48 614 027–61 653 859)	(17 388 026–24 953 299)	(66 277 451–86 347 358)		(4.0–4.5)	(1.7–2.1)	(3.0–3.4)

*PAFs (%) based on DALYs.

†The numbers in parentheses are the 95% uncertainty interval.

‡Percentage of deaths due to occupational risk factors that were due to this risk factor.

§Percentage of DALYs due to occupational risk factors that were due to this risk factor.

¶Particulate matter, gases and fumes (PMGF) and secondhand smoke (SHS) causing COPD.

**Diseases caused by SHS, excluding cancer and COPD.

COPD, chronic obstructive pulmonary disease; DALY, disability-adjusted life year; PAF, population attributable fraction.

In terms of DALYs, there were 76.1 (95% UI 66.3–86.3) million DALYs globally, 3.2% of total DALYs in 2016 (4.2% of those aged 15 years or more), with 72% occurring in males. The risk factors resulting in the greatest overall burden, as measured in DALYs, were injury risk factors (28%) and ergonomic risk factors (20%) (table 1).

The PAFs (based on DALYs) ranged from 3.4% for carcinogens to 19.6% for noise and 26.8% for ergonomic risk factors (table 1). The PAFs based on deaths ranged from 3.9% for carcinogens to 15.7% for PMGF and SHS resulting in COPD.

### Age

**Figure 1:**
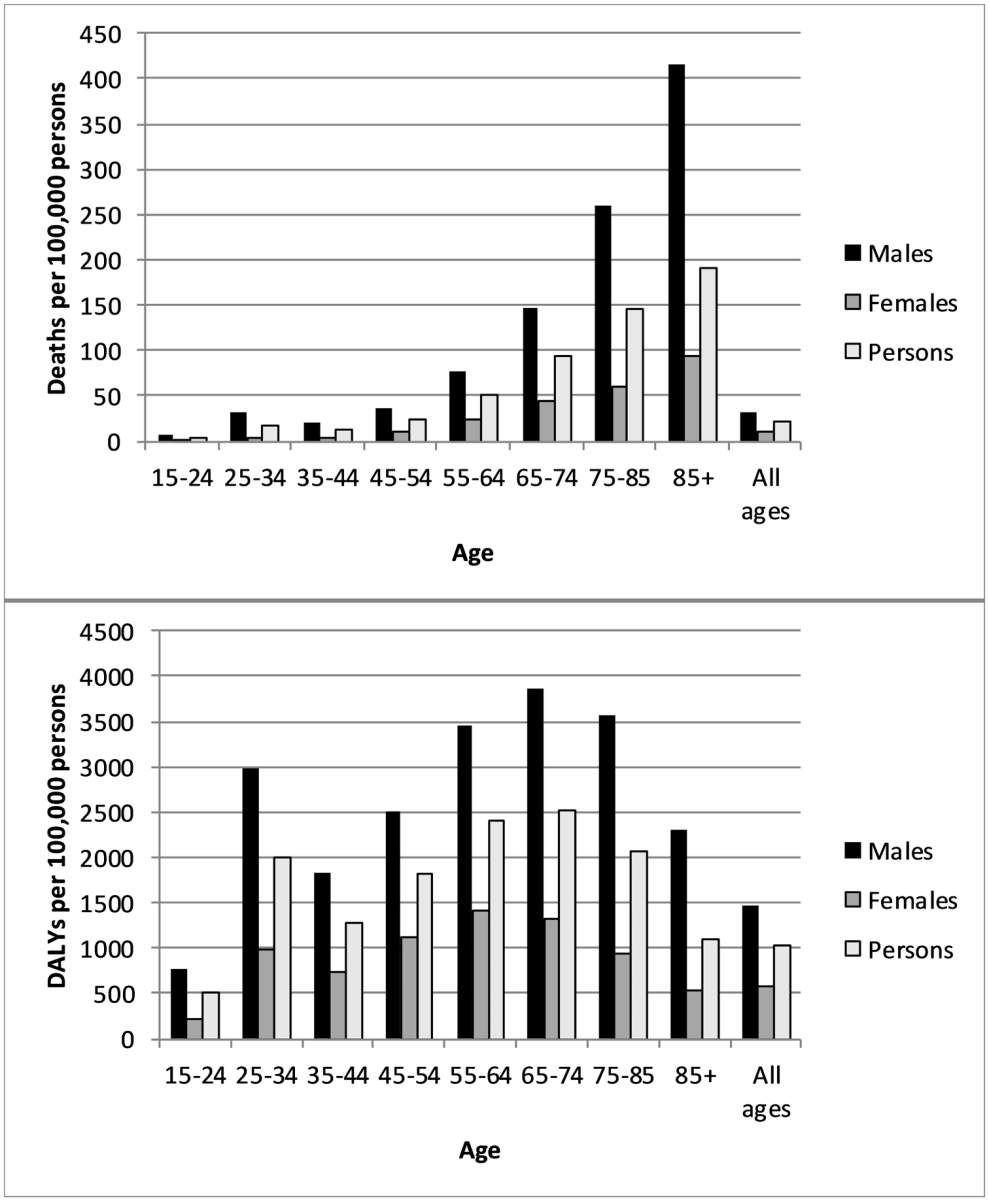
Occupation-attributable deaths and DALYs, by age and sex, 2016 (per 100 000 persons). DALY, disability-adjusted life year.

Seventy-six per cent of deaths but only 39% of DALYs occurred in persons aged 55 years and older. The mortality rate increased with increasing age, more markedly in men than women. For DALYs, from age 35 years the rate increased to age 65–74 years but dropped in older persons (figure 1).

**Figure 2:**
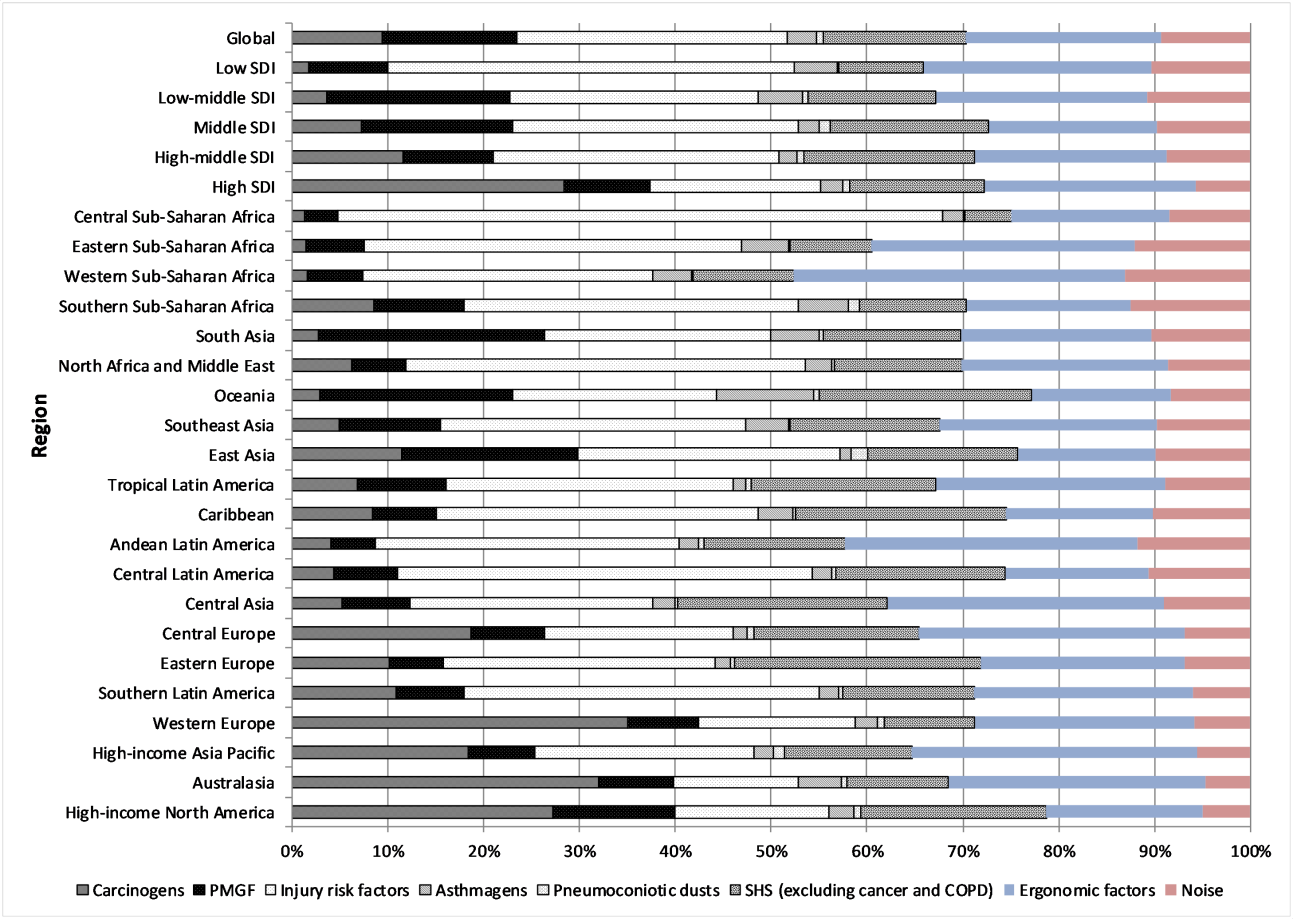
Occupation attributable disability-adjusted life years (DALYs) per capita by region, 2016, per cent. COPD, chronic obstructive pulmonary disease; PMGF, particulate matter, gases and fumes; SDI, sociodemographic index; SHS, secondhand smoke.

### Regions

Carcinogens were responsible for the largest number of deaths in all high-income regions (primarily attributable to asbestos-related cancers, which comprised about 80% of all deaths due to carcinogens in these regions) but only one of the LMI regions (Central Europe). Injury risk factors caused the highest number of deaths in half the other regions. SHS and PMGF also were responsible for a considerable number of deaths (online supplementary table S1). This pattern was similar in terms of DALYs, with additional important contribution from ergonomic risk factors resulting in low back pain. Carcinogens were responsible for the highest burden in three of the four high-income regions; in most other regions, injury risk factors were responsible for the highest or second-highest burden proportions (figure 2, online supplementary table S2).

**Figure 3:**
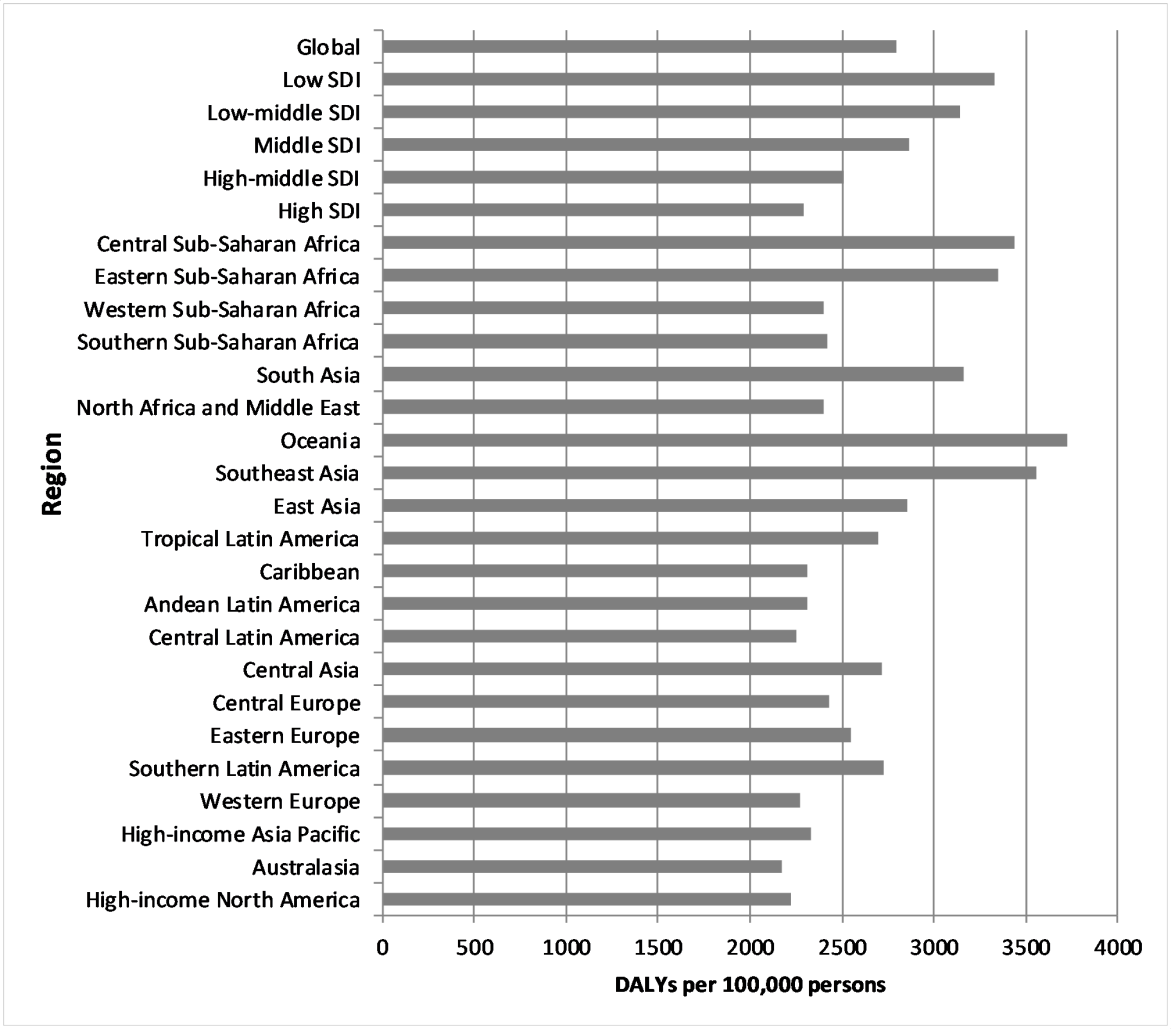
Occupation-attributable DALYs by region, 2016 (per 100 000 persons). DALY, disability-adjusted life year; SDI, sociodemographic index.

The highest number of deaths and DALYs was in the regions with the largest populations—East Asia and South Asia. The highest rates of deaths were in Oceania, East Asia and South Asia, but rates were also high in most high-income regions, whereas for DALYs the highest rates were in Oceania, Southeast Asia and Central sub-Saharan Africa, and high-income regions had the lowest rates. Rates of both were highest in low and low-middle SDI regions (figure 3).

### Changes over time

Between 1990 and 2016 there was a 16% increase in deaths and a 22% increase in DALYs attributable to occupational risk factors. However, rates provide more useful information as they take into account population changes. On a per capita basis, there was an overall decrease of about 31% for deaths and 25% for DALYs, and a considerable percentage decrease in burden, whether measured in deaths or DALYs, for most individual risk factors. The exceptions were SHS-related disease (excluding cancer and COPD), ergonomic risk factors and noise, all of which changed little (table 2).

**Table 2:** Change in global occupation-attributable deaths and DALYs between 1990 and 2016 (per 100 000 persons) (number (95% uncertainty interval) and per cent)

Risk factor	Deaths per 100 000 persons*			DALYs per 100 000 persons*		
	1990†	2016	% change	1990	2016	% change
Carcinogens	15.0	13.5	−9.8	320	272	−14.8
	(12.1–18.1)	(11.0–16.0)		(256–386)	(220–326)	
PMGF+SHS‡	31.0	18.2	−41.3	653	410	−37.1
	(25.8–36.5)	(15.0–21.9)		(545–768)	(346–482)	
Injury risk factors	22.6	12.0	−46.8	1381	779	−43.6
	22.1–23.2)	(11.8–12.3)		(1285–1503)	(708–862)	
Asthmagens	*2.2*	1.4	−36.0	108	85	−21.4
	(1.5–3.0)	(1.0–1.8)		(82–136)	(67–106)	
Pneumoconioses	1.4	0.8	−41.3	36	22	−39.7
	(1.1–2.1)	(0.7–1.0)		(28–53)	(19–26)	
SHS (excluding cancer and COPD)§	12.8	12.6	−1.4	391	417	6.6
	(8.7–17.2)	(8.2–17.3)		(259–532)	(260–582)	
Ergonomic risk factors				574	556	−3.2
				(396–775)	(386–746)	
Noise				246	260	5.4
				(172–338)	(182–358)	
Total	84.9	58.6	−31.1	3709	2800	−24.5
	(71.3–100.1)	(47.7–70.2)		(3023–4490)	(2188–3488)	

*These rates are age and sex standardised.

†The numbers in parentheses are the 95% uncertainty interval.

‡Particulate matter, gases and fumes (PMGF) and secondhand smoke (SHS) causing chronic obstructive pulmonary disease (COPD).

§Diseases caused by SHS, excluding cancer and COPD.

DALY, disability-adjusted life year.

The rates of deaths and DALYs fell across all regions, but the proportionate fall varied considerably between regions, ranging from 15% (Southern Latin America) to 54% (East Asia) for deaths and from 6% (Southern Latin America and Western sub-Saharan Africa) to 43% (East Asia) for DALYs (online supplementary table S3).

## Discussion

This analysis shows that occupational risk factors are responsible for a sizeable proportion of all ill health and injury across the world as measured by deaths (2.8%) and DALYs (3.2%). This is notwithstanding that it was not possible to include all potentially important occupational risk factors (considered below). Injury risk factors and ergonomic risk factors resulting in low back pain were responsible for the biggest overall burden, and PMGF and SHS resulting in COPD were the largest single category in terms of deaths related to occupational exposures. The total burden has risen considerably since 1990. However, when taking into account increases in the population, the per capita burden for all risk factors has actually decreased, though to varying extents. Since the exposure assessments are primarily based on occupation or industry, most of the changes reflect changes in the occupational and industrial distribution of workers as opposed to changes in exposure.

### Other estimates of global occupational burden

The overall methods of the CRA 2000 study^3^ and the current study were similar, but there were some important differences in terms of the exposures and outcomes included, the risk measures (due to updates in the literature), the population numbers, the approach to estimating the population at risk (which was more detailed in the current study than was possible for the CRA 2000 study) and the approach to estimating the prevalence of exposure to asbestos. The CRA study estimated there were 820 000 deaths in 2000 from exposure to occupational risk factors, compared with the estimates in the current study—1.42 million deaths in 2000 and 1.53 million deaths in 2016. The differences arise mainly from the current study estimating twice as many deaths from cancer and 50% more deaths from COPD, and including SHS-associated outcomes apart from cancer and COPD (such as cardiovascular disease). Other estimates of global burden estimate a considerably larger burden of deaths, primarily attributable to a much broader inclusion of exposures and outcomes arising from less restrictive criteria for the strength of the required evidence and not including the equivalent of a counterfactual.^5^

### Implications and uses of the data

There are several clear implications of the results arising from this study. Most importantly, exposures at work remain an important risk factor for a number of outcomes in all regions of the world. The burden arising from carcinogens and noise, and to a lesser extent PMGF and SHS causing COPD, primarily reflects the effects of occupational exposures in past decades, but many of these exposures still occur. Published information does suggest average levels for many exposures have decreased over time, particularly in high-income countries, but much of the data are for inhalational exposures and many instances of high exposure remain.^18–21^ With the transition of much heavy industry and manufacturing from high-income to LMI countries, the adverse experience of high-income countries is likely to be reflected in the future burden for LMI countries unless significant steps are taken to eliminate the problem exposures where possible, and otherwise to control them effectively.^22^ Asbestos is a striking example, where current exposures continue in LMI countries, with a clearly predictable and devastating future burden if current and future exposures are not prevented. In addition, concerns regarding new exposures (eg, nanoparticles) and new exposure situations (eg, silica exposure from artificial stone bench tops) suggest that just controlling the exposures that are included in the current analysis will not eliminate all future ill health arising from occupational exposures.

This study does not provide information on the cost or technological feasibility of eliminating or reducing exposures, or on the uptake of known effective prevention strategies. Thus, it cannot fully inform priorities for resource allocation to decrease the burden from the risk factors considered in this study, assuming that deaths or DALYs preventable per unit expenditure would be taken into account. Nevertheless, the elimination of asbestos exposure is already well recognised as a priority,^23^ and the results presented here serve to further emphasise the importance of this. Excessive noise, many ergonomic exposures, injury risk factors and SHS exposures are clearly eminently preventable in many settings, and better control of them would be expected to lead to significant reduction in the burden of disease arising from occupational exposures affecting the global population. Most of the assessed exposures and the resulting burden are entirely or largely avoidable and in many instances the individual worker has little influence over the level of exposure experienced, emphasising the need for and importance of controls at the organisational and societal level.

### Methodological considerations and limitations

In any study of the type presented here there are uncertainties arising from shortcomings in the data that necessitate significant assumptions. Those made in this study were based on available data and, where necessary, expert opinion of GBD authors. The main uncertainties in the data, and the potential implications of these, are briefly considered below. Where they could be quantified to some extent, they were incorporated into the uncertainty intervals. More detailed consideration of some of these issues is provided in the companion papers.^12 13^

#### Workforce data

The workforce data came from the ILO database, supplemented, where necessary, by information from other sources such as regional surveys. For South Asia (which is predominantly India) and East Asia (which is predominantly China)—the two largest regions—there were limited available data. Some countries did have available data but only for broad industry and occupation categories. Gaps were filled through modelling of the available data for the country and extrapolation from countries in the region for which data were available. The extent and effect of error arising from this extrapolation and modelling is difficult to assess accurately.

A potentially more important source of error was the extent to which the workforce data used for the study included the informal workforce and ‘guest workers’ from other countries. The ILO denominator data are intended to include such workers, but this coverage is very likely to be moderate at best in countries such as India with a very large informal workforce and in countries with many child workers (who were excluded from the current analysis). In some countries and regions this may lead to considerable underestimation of the population at risk and therefore of the burden arising from occupational risk factors. Future iterations of the GBD study will attempt to improve coverage or otherwise adjust for lack of coverage.

#### Population at risk

The carcinogen analyses attempted to take into account the latency between exposure and occurrence of the related cancer when estimating the population at risk, and the fact that workers remain at risk of developing cancer long after they change jobs or leave the workforce, using estimates of workforce turnover and information on life expectancy for each region. The strengths and limitations of this approach, and of the approach to estimating exposure prevalence, are considered elsewhere.^13^ Briefly, the principal uncertainties are that there is a general lack of information on the latency of specific cancers and uncertainty about variation in turnover worldwide.

#### Using industry and occupations as proxy measures for risk factors

A major source of uncertainty stems from the fact that industry and/or occupation were used as the sole or main basis for the risk factor exposure prevalence measures, lacking any data on actual exposure levels in different countries. For carcinogens, exposure prevalence data were available (from CAREX^24^) for individual carcinogens, but only for high-income countries and only on the basis of exposure prevalence, rather than on absolute exposure levels or cumulative exposures. For PMGF, prevalence data were based on limited published information modified by expert opinion. For noise, information was available for absolute exposure levels within different industries, but only for one (high-income) region, and the analysis did not take into account the likely latency of noise-induced hearing loss.

#### Risk measures

The relative risk estimates came primarily from working cohorts of males in high-income countries and from a range of calendar time periods. The workers within the cohorts had a range of exposures in terms of length of exposure and intensity of exposure. The length of follow-up varied. Therefore, there will have been some mismatch between the relative risk estimates used and the exposure circumstances to which they have been applied, particularly for LMI countries. Nevertheless, the measures used were considered the most appropriate available. A major gap in available relative risks was the lack of separate risk estimates for women and/or persons of different age for nearly all risk–outcome pairs.

#### Exclusion of exposures and outcomes

There are many occupational exposures that are probably related to adverse health outcomes that were not included in the study reported here. These include International Agency for Research on Cancer Group 2A carcinogens (‘*probably carcinogenic to humans*’) associated with occupational exposures; infections arising from occupation; occupational exposures (apart from SHS) linked to ischaemic heart disease; pesticides; psychological and psychosocial stresses; work organisation factors; and occupational exposures leading to musculoskeletal disorders (other than those affecting the lower back). These were excluded because there did not appear to be reliable data on one or both of the exposures or the associated risk measures; and/or because the level of evidence supporting a causal association was not considered sufficient. If a causal connection was accepted, many of these exposure–outcome pairs would be expected to result in a considerable number of deaths and/or DALYs. Their exclusions might therefore be expected to have resulted in a considerable underestimation of the total burden arising from occupational risk factors. The most important exclusions in terms of numbers of deaths are likely to be shift work, with breast cancer the associated outcome; occupational exposure to the ultraviolet component of sunlight, leading to skin cancer; exposures such as psychosocial factors resulting in ischaemic heart disease; and possibly occupational exposure to herbicides and insecticides in relation to non-Hodgkin’s lymphoma. In terms of DALYs, psychological and psychosocial stresses leading to adverse mental health outcomes, occupational exposures leading to musculoskeletal disorders and heat-related disorders worsened by climate change would be expected to result in considerable burden.

#### Injury risk factors and injuries

Our estimates assumed the percentage (by age group and sex) of non-fatal injuries that were occupational equalled the percentage of fatal injuries that were occupational. Lack of data precluded a more refined estimation approach, but aggregate US data suggest that the assumption may severely underestimate non-fatal injuries (eg, in 1999, at ages 15–64, US occupational injuries accounted for 5.7% of injury deaths and 19.9% of medically attended injuries^25 26^). Also, note that the estimates are likely to omit occupational injuries (and diseases) of sex workers and trafficked workers (who experience high rates of violent injury, sexually transmitted diseases and unintentional injury^27 28^) and most active-duty military injury deaths.

#### Overall effect of the limitations

The extent and direction of bias potentially arising from the major limitations is difficult to estimate with confidence for most of these limitations. Exclusion of some of the informal workforce, exclusion of some potentially important exposures and outcomes and use of fatal injury PAFs for non-fatal injury would all be expected lead to an underestimation of the associated burden. The approach used to estimate the population at risk and using industry and occupation as proxy measures for the prevalence of exposure to risk factors could result in bias in either direction. The use of CAREX for LMI countries might be expected to lead to an underestimation of exposure prevalence, and the use of relative risks from high-income countries for all countries might be expected to lead to an underestimation of risk in LMI countries, any error from these sources being expected to lead to an underestimation of the burden in LMI countries.

### Recommendations for further work

This analysis has identified several areas where further research could improve the accuracy and usefulness of the burden estimates. Several potentially important risk factor–outcome pairs were not included for various reasons, as mentioned earlier. Ongoing appraisal of the literature is being undertaken to address some of these, but better evidence regarding causal link will be required before decisions to include or exclude can be made confidently. There is also considerable scope for improvement in the assessment of the prevalence of exposure to those risk factors that were included in this study. Maximising the inclusion of the unofficial workforce is important to overcome a potentially important source of underestimation of the exposed population. Data to support these approaches will be sought for upcoming GBD iterations.

## Conclusion

Occupational exposures are an important cause of largely preventable disease and injury burden in all regions of the world. Changes in the burden over the last two decades, as highlighted through burden of disease studies, have varied considerably among risk factors and regions. The results provide guidance to assess priorities, as well as justification for prevention and control initiatives.

10.1136/oemed-2019-106008.supp2 Supplementary data
