# Supplementary material for: Global and regional burden of disease and injury in 2016 arising from occupational exposures: a systematic analysis for the Global Burden of Disease Study 2016
Source: Occup Environ Med. 2020 Feb 13;77(3):133–41. doi: 10.1136/oemed-2019-106008 (PMC7035694; doi:10.1136/oemed-2019-106008)
Supplement: Supplementary data [file oemed-2019-106008supp001.pdf]

Global and regional burden of disease and injury in 2016 arising from occupational exposures: a systematic analysis for the Global Burden of Disease Study 2016

GBD 2016 Occupational Risk Factors Collaborators

SUPPLEMENTARY MATERIAL

APPENDIX 1: DETAILED INFORMATION ON METHODOLOGY

Carcinogens and cancer

The exposure information for carcinogens was based primarily on the CAREX (Carcinogen Exposure) database, which provides industry-specific information on prevalence of exposure to various carcinogens in Western Europe for 1990 to 1993[1]. This was used as the basis for estimating the proportion of workers within an industry who were exposed to each of the relevant carcinogens. These proportions were distributed between “high” and “low” exposure based on information on exposure prevalence in high-income (countries in the Australasia, high-income North America, Western Europe, and high-income Asia Pacific regions) and low- and middle-income (LMI) countries (all other countries) from identified relevant cohort studies[2-8]. On the basis of this information, the CAREX prevalences were distributed 10:90 (high:low) for high-income countries and 50:50 (high:low) for LMI countries. Separate relative risks were estimated for high and low exposure.

Fourteen separate exposures were included in the carcinogens analysis and linked to seven cancer types. Selection of exposure-cancer pairs for inclusion was based on information in International Agency for Research on Cancer (IARC) Monographs 1-106 and expert assessments regarding exposure prevalence and availability of exposure data. We included all IARC Group 1 (definite human) carcinogens with relevant occupational exposure circumstances, a non-trivial number of cases, non-trivial exposure levels, a non-trivial proportion of persons exposed, and available exposure data; and all associated outcomes for which there was sufficient epidemiological evidence. The included carcinogen-outcome pairs are documented in the companion paper on occupational carcinogens[9]. Our primary analysis did not include any IARC Group 2A exposures (probable carcinogens), although these are addressed to some extent in the companion paper.

Age-specific numbers ever exposed during the risk-exposure period were estimated accounting for latency of the cancers and for workers formerly employed in the industry still being at risk. To accomplish this,

occupational turnover estimates (OTs) based on a risk-exposure period defined by cancer latency (10-50 years for solid tumours [long latency], 0-20 years for haematopoietic cancers [short latency]), annual staff turnover estimates, and normal life expectancy were developed and applied to the original prevalence data. Separate estimates were provided for men and for women, and for the solid tumours and for haematopoietic cancers, for 2016. Separate life tables (based on a representative country in each region) were used to estimate the OTs by region. Further detail on the approach used is available elsewhere[9 10].

A different approach was taken to estimate the proportion of persons ever exposed to asbestos. Rates of malignant mesothelioma were used to estimate the past prevalence of exposure to asbestos in each country. This Asbestos Impact Ratio approach was analogous to the Smoking Impact Ratio approach described elsewhere[11]. The relevant companion paper provides more detail on this and the methods used for the occupational carcinogen analysis[9]. The comparison for each carcinogen-specific analysis was no exposure above background to the relevant carcinogen.

Particulate matter, gases and fumes, and chronic obstructive pulmonary disease

Industry was used as a proxy for exposure to particulate matter, gases and fumes (PMGF) because we identified no suitable and valid data sources at a country or global level of exposure to PMGF, either singly or to PMGF as a group. Current industry was used as the basis of exposure estimates, but the estimates of proportions exposed within each industry attempted to take into account past exposure (to estimate ever exposed), given that both past and current exposure appear to increase the risk of chronic obstructive pulmonary disease (COPD). Estimates of proportion exposed at lower and higher levels were based on sparse published data and expert opinion by GBD authors. Information on risk was obtained by conducting a systematic review of international literature and meta-analysis of relevant results. Relative risks in these studies were for COPD greater than or equal to GOLD stage II (defined as requiring non-reversibility after using bronchodilators for provocation, a forced expiratory volume in one second/forced vital capacity (FEV_1/FVC) ratio of less than 0.70 and an FEV_1 of less than 80% predicted)[12]. Relative risk estimates were used for an overall “lower” level and an overall “higher” level of exposure to the agents of concern. The

reference group was persons not working and persons working in trade, finance, or service industries. Further information on the methods is available in the companion paper on airborne risk factors[13].

Asthmagens and asthma

Exposure for asthmagens was based on the current occupation distribution in each country because there were no suitable and valid data sources at a country or global level describing exposure to the wide range of occupational asthmagens. All relative risk information except that for agricultural occupations came from a study by Karjalainen and coworkers, a comprehensive national population study of incident asthma [14 15]. Relative risks for agricultural occupations were based on a separate study by Kogevinas and coworkers[16], with an inverse variance-weighted estimate obtained using the separate estimates for “farmers” and “agricultural” workers provided in the paper. This information was used because the results were thought to be more generalizable to agriculture in the rest of the world, especially the LMI regions. Separate risks were available and used for males and females (except for agricultural operations), although the sex-specific risks were similar and within the limits of random variation. The same relative risks were used for all age groups. The referent group (used as the counterfactual) was persons not working and administrative workers. Further information on methods is available in the companion paper on airborne risk factors[13].

Pneumoconioses

Pneumoconioses were estimated as part of the envelope (the total number of cases) of disease component of the GBD, rather than using the attributable fraction approach. The methods used are described elsewhere[17]. The attributable fraction is essentially 100% because virtually all pneumoconioses arise as a result of occupational exposure. Separate estimates were available for silicosis, asbestosis, and coal workers’ pneumoconiosis (CWP), with the remaining cases grouped under an “other pneumoconiosis” category.

Noise and noise-induced hearing loss

Current industry was used as a proxy for exposure to occupational noise because of insufficient useable exposure data at a country or global level. Australian national data on noise exposure in various industries (sampled across a range of tasks) provided the basis for the mean and standard deviation of noise exposure in each industry[18]. This information was in turn used to estimate the proportion of workers exposed at "low" levels of noise (85-<90dB) and "high" levels of noise (90dB or higher). For the presented analysis, no account was made of the use of hearing protection, except for the mining industry, where mean exposure levels were decreased by 3dB (based on expert opinion) to take this into account. The proportions were modified for LMI countries, to take into account the likely higher exposure levels due to the less extensive use of noise controls. For LMI countries, the mean exposure was estimated to be 3dB higher (over an entire shift – double the noise level of high-income countries) in 1990, and 1.8dB higher (50% increase in the noise level of developed countries) in 2016 (based on expert opinion).

The outcome factor was hearing loss at 41dB or greater. Relative risks for high and low exposure were obtained in a two-step method. First, the absolute excess proportions of persons with hearing loss at 41dB or greater due to occupational exposures at 85-90dB, and at greater than 90dB, was obtained, separately for different ages, from Nelson and coworkers[19]. Information on background prevalence of hearing loss at 41dB or greater was obtained from population surveys in the United Kingdom[20] and Australia[21]. The same excess and population prevalences were used for all countries. The relative risk (RR) was then estimated as RR = 1+ excess prevalence of hearing loss/background prevalence of hearing loss. This was calculated separately for the age ranges used in the relevant publications and then adjusted to fit the GBD age ranges.

For example, for ages 50 to 59 years, the absolute excess proportion of persons with hearing loss at 41dB or greater due to occupational noise exposure at greater than 90dB was 0.169. The background prevalence of hearing loss at 41dB or greater was 0.037. Therefore, the relative risk for this age group was 1+(0.169/0.037) = 5.6.

The referent group was persons exposed only at background noise levels. Further information on methods is available in our companion paper on occupational noise[22].

Ergonomic risk factors and low back pain

Current occupation was used as a proxy for ergonomic risk factors associated with occupation, as described in detail elsewhere[23]. The occupations were grouped to be consistent with the exposure data in the studies that provided risk information. Information on the relative risk of low back pain was obtained from a systematic review of international literature and meta-analysis of relevant results, as described previously[23]. The same relative risk estimates were used for males and females and for all age groups. The referent group was persons not working and clerical workers.

Injury risk factors and injury

“Injury” was defined as any injury to a worker due to work-related exposures and which would have warranted some type of health care in a system with full access to health care[24], excluding self-harm and injuries sustained driving between home and work or vice versa. The approach to estimating injuries did not use population attributable fraction methods (although we did calculate PAFs secondarily after having estimated the proportion of work-related injuries). Instead, the number of fatal occupational injuries was estimated by applying industry-specific fatal injury rates taken directly from the ILO database[25] (by country where available and modeled using covariates and rates in geographically and demographically similar countries when country-specific data were not available) to the estimated industry-specific population in each country. The same rates were used for all ages and both sexes, but age- and sex-specific PAFs were estimated by dividing the work-related fatal injuries by the total fatal injuries in the relevant age-sex group. The injuries were distributed across different injury causes using a custom set of injuries that were selected for each industry a priori, based on previous published work[26 27]. Lacking suitable data, non-fatal injury PAFs could not be estimated directly. Instead, non-fatal injury PAFs were assumed to be the same as the fatal injury PAFs. The comparison was zero work-related injuries.

Second-hand smoke

Second-hand smoke was included as a risk factor for several different types of outcomes, consistent with the approach taken in the GBD overall. The relevant outcomes were breast cancer, lung cancer, ischaemic heart disease, stroke, lower respiratory tract infection and diabetes mellitus. Information on exposure prevalence was as described above for carcinogens, except that separate “high” and “low” prevalences were not used for the non-cancer outcomes. Further information on the relative risk measures used is in the main GBD risk factors paper[28].

Table S1 Global occupational-attributable deaths by risk factor and region, 2016 – percent

Region	Number	Carcinogens %	PMGF[1] %	Injury risk factors %	Asthmagens %	Pneumoconiotic dusts %	SHS (excluding cancer and COPD)[2] %	Total %
Global	1,532,431	22.8	30.0	21.7	2.5	1.4	21.7	100.0
Low SDI[3]	91,849	4.7	22.3	48.4	5.6	0.6	18.4	100.0
Low-middle SDI	377,822	8.0	41.4	22.7	5.7	1.1	21.1	100.0
Middle SDI	535,931	14.2	34.7	24.4	1.6	1.5	23.6	100.0
High-middle SDI	235,497	25.3	23.8	21.7	0.6	1.2	27.4	100.0
High SDI	291,332	61.2	14.2	7.1	0.3	1.9	15.3	100.0
Central sub-Saharan Africa	13,684	3.6	9.7	74.2	2.3	0.5	9.7	100.0
Eastern sub-Saharan Africa	39,607	4.6	19.5	48.7	6.2	0.6	20.4	100.0
Western sub-Saharan Africa	25,517	5.4	18.7	40.9	5.5	0.2	29.3	100.0
Southern sub-Saharan Africa	10,352	21.8	20.4	33.7	4.6	2.5	16.9	100.0
South Asia	340,754	5.6	46.1	19.4	6.2	1.2	21.5	100.0
North Africa and Middle East	71,234	16.6	13.8	44.0	1.9	0.6	23.1	100.0
Oceania	2,508	5.1	35.3	18.0	11.6	1.2	28.8	100.0
Southeast Asia	128,695	12.2	21.8	32.7	5.7	0.2	27.4	100.0
East Asia	424,775	18.9	40.3	19.1	0.3	2.1	19.4	100.0
Tropical Latin America	41,640	16.3	23.9	26.1	0.4	1.1	32.3	100.0
Caribbean	6,895	18.9	17.3	25.7	2.0	0.3	35.8	100.0
Andean Latin America	6,665	13.1	18.6	35.9	0.5	1.8	30.1	100.0
Central Latin America	34,653	10.5	20.3	41.0	0.5	0.7	26.9	100.0
Central Asia	13,008	12.3	19.9	22.8	1.3	0.5	43.2	100.0
Central Europe	25,391	41.3	18.2	9.8	0.2	1.0	29.5	100.0
Eastern Europe	48,709	21.5	13.1	21.4	0.4	0.6	43.1	100.0
Southern Latin America	13,465	25.9	21.1	28.8	0.3	1.5	22.4	100.0
Western Europe	133,939	69.0	13.6	5.2	0.1	2.1	10.0	100.0
High-income Asia Pacific	42,184	55.2	12.2	10.7	0.3	3.8	17.8	100.0
Australasia	7,566	68.3	13.8	4.7	0.3	1.4	11.5	100.0
High-income North America	101,190	55.5	16.5	7.3	0.4	1.1	19.2	100.0

1: Includes chronic obstructive pulmonary disease (COPD) caused by second-hand smoke (SHS) as well as by particulate matter, gases and fumes (PMGF).
2: Diseases caused by (SHS), excluding cancer and COPD.
3: SDI – Socio-demographic index.

Table S2 Global occupational-attributable DALYs by risk factor and region, 2016 – percent[1]

Region	Number	Carcinogens %	PMGF[2] %	Injury risk factors %	Asthmagens %	Pneumoconiotic dusts %	SHS (excluding cancer and COPD)[3] %	Ergonomic factors %	Noise %	Total %
Global	76,133,534	9.5	14.0	28.2	3.1	0.8	14.9	20.3	9.3	100.0
Low SDI[4]	6,669,646	1.8	8.3	42.5	4.4	0.2	8.8	23.8	10.3	100.0
Low-middle SDI	20,830,127	3.7	19.0	25.9	4.7	0.5	13.4	22.0	10.8	100.0
Middle SDI	25,985,832	7.3	15.7	29.8	2.3	1.1	16.6	17.6	9.7	100.0
High-middle SDI	12,077,026	11.6	9.4	29.8	1.9	0.7	17.8	20.0	8.7	100.0
High SDI	10,570,902	28.4	9.0	17.8	2.3	0.8	14.0	22.1	5.6	100.0
Central sub-Saharan Africa	991,648	1.3	3.6	63.0	2.2	0.2	4.9	16.5	8.5	100.0
Eastern sub-Saharan Africa	3,314,178	1.5	6.1	39.4	4.9	0.2	8.5	27.4	12.1	100.0
Western sub-Saharan Africa	2,487,946	1.6	5.8	30.3	4.1	0.1	10.5	34.6	13.0	100.0
Southern sub-Saharan Africa	586,149	8.6	9.4	34.9	5.2	1.0	11.2	17.1	12.5	100.0
South Asia	17,845,895	2.8	23.7	23.5	5.0	0.5	14.3	19.8	10.4	100.0
North Africa and Middle East	4,659,997	6.2	5.7	41.6	2.8	0.3	13.3	21.5	8.6	100.0
Oceania	122,968	3.0	20.1	21.2	10.1	0.7	22.1	14.4	8.4	100.0
Southeast Asia	8,105,344	4.9	10.6	31.8	4.5	0.2	15.5	22.7	9.8	100.0
East Asia	17,573,844	11.4	18.4	27.4	1.1	1.7	15.6	14.4	9.9	100.0
Tropical Latin America	2,240,758	6.9	9.1	30.0	1.3	0.6	19.3	23.9	8.9	100.0
Caribbean	363,640	8.5	6.7	33.5	3.7	0.2	22.0	15.3	10.2	100.0
Andean Latin America	473,819	4.1	4.7	31.6	2.1	0.5	14.8	30.5	11.7	100.0
Central Latin America	2,019,282	4.4	6.6	43.4	1.9	0.5	17.7	14.9	10.7	100.0
Central Asia	848,786	5.2	7.1	25.4	2.3	0.3	21.9	28.8	9.1	100.0
Central Europe	1,279,590	18.7	7.7	19.6	1.5	0.7	17.2	27.7	6.9	100.0
Eastern Europe	2,434,958	10.1	5.8	28.3	1.7	0.3	25.7	21.2	6.9	100.0
Southern Latin America	687,307	10.9	7.2	36.9	2.1	0.5	13.6	22.9	6.0	100.0
Western Europe	4,429,822	35.1	7.3	16.3	2.3	0.8	9.4	22.9	5.8	100.0
High-income Asia Pacific	1,909,424	18.4	7.0	22.9	2.0	1.1	13.3	29.8	5.5	100.0
Australasia	268,135	32.1	7.8	13.0	4.5	0.5	10.6	26.8	4.7	100.0
High-income North America	3,490,045	27.3	12.7	16.2	2.5	0.8	19.3	16.3	5.0	100.0

1: DALY=Disability-adjusted life year.
2: Includes chronic obstructive pulmonary disease (COPD) caused by second-hand smoke (SHS) as well as by particulate matter, gases and fumes (PMGF).
3: Diseases caused by SHS, excluding cancer and COPD.
4 SDI – Socio-demographic index.

Table S3 Change in global occupational-attributable deaths and DALYs between 1990 and 2016 by region (per 100,000 persons and percent)[1]

Region	Deaths per 100,000 persons[2] 1990	2016	% change	DALYs per 100,000 persons[2] 1990	2016	% change
Global	34.9	22.4	-35.8	1,430	1,037	-27.5
Low SDI[3]	34.0	23.4	-31.2	1,756	1,334	-24.0
Low-middle SDI	40.1	26.8	-33.2	1,647	1,176	-28.6
Middle SDI	45.8	25.8	-43.6	1,638	1,119	-31.7
High-middle SDI	29.4	17.4	-40.7	1,257	867	-31.0
High SDI	25.8	17.1	-33.5	1,054	783	-25.7
Central sub-Saharan Africa	25.5	20.7	-19.0	1,523	1,312	-13.9
Eastern sub-Saharan Africa	31.6	20.6	-34.9	1,813	1,344	-25.9
Western sub-Saharan Africa	16.0	13.3	-17.0	1,027	964	-6.2
Southern sub-Saharan Africa	27.9	20.4	-27.0	1,303	949	-27.2
South Asia	44.0	29.2	-33.6	1,745	1,246	-28.6
North Africa and Middle East	25.5	16.6	-34.8	1,351	914	-32.3
Oceania	48.7	38.3	-21.4	1,805	1,482	-17.9
Southeast Asia	28.3	23.6	-16.4	1,479	1,288	-12.9
East Asia	59.9	27.6	-53.9	1,814	1,036	-42.9
Tropical Latin America	28.6	20.6	-28.0	1,248	1,005	-19.5
Caribbean	18.9	15.3	-19.0	934	790	-15.5
Andean Latin America	20.7	13.9	-32.9	996	883	-11.4
Central Latin America	21.5	16.2	-24.4	1,079	843	-21.9
Central Asia	25.0	17.9	-28.3	1,287	1,027	-20.2
Central Europe	21.1	14.4	-31.7	1,134	845	-25.5
Eastern Europe	24.0	16.7	-30.5	1,200	912	-24.0
Southern Latin America	22.4	19.0	-15.3	1,066	1,003	-5.9
Western Europe	25.5	17.4	-31.7	992	768	-22.6
High-income Asia Pacific	21.3	11.9	-44.0	1,191	796	-33.2
Australasia	26.8	18.4	-31.3	963	759	-21.1
High-income North America	28.0	19.8	-29.5	1,016	768	-24.4

1: DALY=Disability-adjusted life year.
2: These rates are age and sex-standardised and based on all persons, not just persons 15 years and over.
3: SDI – Socio-demographic index.

REFERENCES FOR SUPPLEMENTARY MATERIAL

1. Kauppinen T, Toikkanen J, Pedersen D, et al. Occupational exposure to carcinogens in the European Union. *Occupational and Environmental Medicine* 2000;57(1):10-8.

2. Dosemeci M, McLaughlin J, Chen J, et al. Historical total and respirable silica dust exposure levels in mines and pottery factories in China. *Scandinavian Journal of Work, Environment and Health* 1995;21(Suppl 2):39–43.

3. Partanen T, Jaakkola J, Tossavainen A. Silica, silicosis and cancer in Finland. *Scandinavian Journal of Work, Environment and Health* 1995;21(Suppl 2):84–86.

4. Myers J, Lewis P, Hofmeyr W. Respiratory health of brickworkers in Cape Town, South Africa: Background, aims, and dust exposure determinations. *Scandinavian Journal of Work, Environment and Health* 1989;15(3):180–87.

5. National Institute for Occupational Safety and Health (NIOSH). Work-related Lung Disease Surveillance Report 1999. Cincinnati: U.S. Department of Health and Human Services, National Institute for Occupational Safety and Health, 1999.

6. National Institute for Occupational Safety and Health (NIOSH). Injuries, illnesses, and hazardous exposures in the mining industry, 1986-1995: A surveillance report. Washington, DC: U.S. Department of Health and Human Services, National Institute for Occupational Safety and Health., 2000: .

7. Rees D, Cronje R, du Toit R. Dust exposure and pneumoconiosis in a South African pottery. 1. Study objectives and dust exposure. *British Journal of Industrial Medicine* 1992;49(7):459–64.

8. Yin S, Li Q, Liu Y, et al. Occupational exposure to benzene in China. *British Journal of Industrial Medicine* 1987;44(3):192–95.

9. GBD 2016 Occupational Carcinogens Collaborators. Global and regional burden of cancer in 2016 arising from occupational exposure to carcinogens: a systematic analysis for the Global Burden of Disease Study 2016. *Occupational and Environmental Medicine* 2019:Submitted.

10. Rushton L, Hutchings S, Fortunato L, et al. Occupational cancer burden in Great Britain. *British Journal of Cancer* 2012;107(Suppl 1):S3-S7.

11. Peto R, Boreham J, Lopez A, et al. Mortality from tobacco in developed countries: indirect estimation from national vital statistics. *Lancet* 1992;339(8804):1268–78.

12. Vestbo J, Hurd S, Agustí A, et al. Global strategy for the diagnosis, management, and prevention of chronic obstructive pulmonary disease: GOLD executive summary. *American Journal of Respiratory and Critical Care Medicine* 2013;187(4):347-65.

13. GBD 2016 Occupational Respiratory Risk Factors Collaborators. Global and regional burden of chronic respiratory disease in 2016 arising from non-infectious airborne occupational exposures: a systematic analysis for the Global Burden of Disease Study 2016. *Occupational and Environmental Medicine* 2019:Submitted.

14. Karjalainen A, Kurppa K, Martikanen R, et al. Exploration of asthma risk by occupation -extended analysis of an incidence study of the Finnish population. *Scandinavian Journal of Work, Environment and Health* 2002;28:49–57.

15. Karjalainen A, Kurppa K, Martikanen R, et al. Work is related to a substantial portion of adult-onset asthma incidence in the Finnish population. *American Journal of Respiratory and Critical Care Medicine* 2001;164:565–68.
16. Kogevinas M, Anto J, Sunyer J, et al. Occupational asthma in Europe. *Lancet* 1999;353:1750–54.
17. GBD 2016 Mortality Collaborators. Global, regional, and national under-5 mortality, adult mortality, age-specific mortality, and life expectancy, 1970-2016: a systematic analysis for the Global Burden of Disease Study 2016. *Lancet* 2017;390(10100):1084-150. doi: 10.1016/S0140-6736(17)31833-0
18. Williams W. The epidemiology of noise exposure in the Australian workforce. *Noise & Health* 2013;15(66):326-31.
19. Nelson D, Nelson R, Concha-Barrientos M, et al. The global burden of occupational noise-induced hearing loss. *American Journal of Industrial Medicine* 2005;48(6):446-58.
20. Davis A. The prevalence of hearing impairment and reported hearing disability among adults in Great Britain. *International Journal of Epidemiology* 1989;18: 911-17.
21. Wilson D, Walsh P, Sanchez L, et al. The epidemiology of hearing impairment in an Australian adult population. *International Journal of Epidemiology* 1999;28:247-52.
22. Driscoll T, Williams W, Hogan A, et al. Global burden of hearing loss arising from occupational exposure to noise. *Occupational and Environmental Medicine* 2017:Submitted.
23. Driscoll T, Jacklyn G, Orchard J, et al. The global burden of occupationally related low back pain: estimates from the Global Burden of Disease 2010 study. *Annals of Rheumatic Disease* 2014;73(6):975-81.
24. GBD 2016 Disease and Injury Incidence and Prevalence Collaborators. Global, regional, and national incidence, prevalence, and years lived with disability for 328 diseases and injuries for 195 countries, 1990-2016: a systematic analysis for the Global Burden of Disease Study 2016. *Lancet* 2017;390(10100):1211-59. doi: 10.1016/S0140-6736(17)32154-2
25. International Labour Office. ILOSTAT database. In: ILO, ed. Geneva: ILO, 2015.
26. Driscoll T. The epidemiology of work-related fatalities in Australia. University of Sydney, 2003.
27. Driscoll T, Mitchell R, Mandryk J, et al. Work-related fatalities in Australia, 1989 to 1992: an overview. *Journal of Occupational Health and Safety - Australia New Zealand* 2001;17(1):45-66.
28. GBD 2016 Risk Factors Collaborators. Global, regional, and national comparative risk assessment of 84 behavioural, environmental and occupational, and metabolic risks or clusters of risks, 1990-2016: a systematic analysis for the Global Burden of Disease Study 2016. *Lancet* 2017;390(10100):1345-422. doi: 10.1016/S0140-6736(17)32366-8
