# Supplementary material for: Global and regional burden of disease and injury in 2016 arising from occupational exposures: a systematic analysis for the Global Burden of Disease Study 2016
Source: Occup Environ Med. 2020 Feb 13;77(3):133–41. doi: 10.1136/oemed-2019-106008 (PMC7035694; doi:10.1136/oemed-2019-106008)
Supplement: Supplementary data [file oemed-2019-106008supp002.pdf]

GBD 2016 Occupational Risk Factors Collaborators

Tim Driscoll[1], Lesley Rushton[2], Sally J Hutchings[3], Kurt Straif[4], Kyle Steenland[5], Degu Abate[6], Cristiana Abbafati[7], Dilaram Acharya[8,9], Oladimeji M Adebayo[10], Mahdi Afshari[11], Tomi Akinyemiju[12,13], Fares Alahdab[14], Mina Anjomshoa[15,16], Carl Abelardo T. Antonio[17,18], Olatunde Aremu[19], Zerihun Ataro[6], Beatriz Paulina Ayala Quintanilla[20,21], Joseph Adel Mattar Banoub[22], Suzanne Lyn Barker-Collo[23], Till Winfried Bärnighausen[24,25], Lope H Barrero[26], Neeraj Bedi[27,28], Masoud Behzadifar[29], Meysam Behzadifar[30], Fernando G. Benavides[31], Mircea Beuran[32,33], Krittika Bhattacharyya[34,35], Ali Bijani[36], Rosario Cárdenas[37], Juan J Carrero[38], Félix Carvalho[39,40], Carlos A Castañeda-Orjuela[41,42], Ester Cerin[43,44], Cyrus Cooper[45,46], Lalit Dandona[47,48], Rakhi Dandona[47,48], Anh Kim Dang[49], Ahmad Daryani[50], Beruk Berhanu Desalegn[51], Samath Dhamminda Dharmaratne[48,52], Eleonora Dubljanin[53], Ziad El-Khatib[54], Sharareh Eskandarieh[55], Mohammad Fareed[56], Andre Faro[57], Seyed-Mohammad Fereshtehnejad[58,59], Eduarda Fernandes[60], Irina Filip[61,62], Florian Fischer[63], Takeshi Fukumoto[64,65], Silvano Gallus[66], Teklu Gebrehiwo Gebremichael[67], Kebede Embaye Gezae[68], Tiffany K Gill[69], Bárbara Niegia Garcia Goulart[70], Ayman Grada[71], Yuming Guo[72,73], Rahul Gupta[74,75], Arvin Haj-Mirzaian[76,77], Arya Haj-Mirzaian[76,78], Randah R Hamadeh[79], Samer Hamidi[80], Behrooz Hamzeh[81], Hadi Hassankhani[82,83], Devan M Hawkins[84,85], Simon I. Hay[48,86], Mohamed I Hegazy[87], Andualem Henok[88], Chi Linh Hoang[89], Michael K. Hole[90], Enayatollah Homaie Rad[91,92], Naznin Hossain[93,94], Mostafa Hosseini[95], Sorin Hostiuc[96,97], Guoqing Hu[98], Olayinka Stephen Ilesanmi[99], Seyed Sina Naghibi Irvani[100,101], Sheikh Mohammed Shariful Islam[102,103], Mihajlo Jakovljevic[104,105], Ravi Prakash Jha[106], Jost B. Jonas[107,108], Zahra Jorjoran Shushtari[109], Jacek Jerzy Jozwiak[110,111], Mikk Jürisson[112], Amaha Kahsay[113], Manoochehr Karami[114,115], Narges Karimi[116,117], Amir Kasaeian[118,119], Norito Kawakami[120], Yousef Saleh Khader[121], Ejaz Ahmad Khan[122], Jagdish Khubchandani[123], Yun Jin Kim[124], Adnan Kisa[125,126], Soewarta Kosen[127], Parvaiz A Koul[128], Ai Koyanagi[129,130], Barthelemy Kuate Defo[131,132], G Anil Kumar[47], Manasi Kumar[133,134], Faris Hasan Lami[135], Arman Latifi[136], James Leigh[137], Miriam Levi[138,139], Shanshan Li[72], Shai Linn[140], Jaifred Christian F Lopez[141,142], Raimundas Lunevicius[143,144], Narayan Bahadur Mahotra[145], Marek Majdan[146], Reza Malekzadeh[147,148], Mohammad Ali Mansournia[95], Benjamin Ballard Massenburg[149], Varshil Mehta[150], Addisu Melese[151], Ziad A Memish[152,153], Walter Mendoza[154], Getnet Mengistu[155,156], Atte Meretoja[157,158], Tuomo J Meretoja[159], Tomislav Mestrovic[160,161], Bartosz Miazgowski[162], Tomasz Miazgowski[163], Ted R Miller[164,165], GK Mini[166,167], Erkin M Mirrakhimov[168,169], Babak Moazen[170,171], Naser Mohammad Gholi Mezerji[172], Shafiu Mohammed[24,173], Farnam Mohebi[101,174], Ali H Mokdad[48,86], Mariam Molokhia[175], Lorenzo Monasta[176], Yoshan Moodley[177], Mahmood Moosazadeh[178], Ghobad Moradi[179,180], Maziar Moradi-Lakeh[181], Lidia Morawska[182], Shane Douglas Morrison[183], Seyyed Meysam Mousavi[16], Ghulam Mustafa[184,185], Farid Najafi[186], Vinay Nangia[187], Ionut Negoi[33,32], Ruxandra Irina Negoi[188,189], Subas Neupane[190], Cuong Tat Nguyen[49], Trang Huyen Nguyen[89], Molly R Nixon[48], Richard Ofori-Asenso[191,192], Felix Akpojene Ogbo[193], Andrew T Olagunju[194,195], Bolajoko Olubukunola Olusanya[196], Stanislav S Otstavnov[197,198], Mahesh P A[199], Songhomitra Panda-Jonas[200], Eun-Kee Park[201], Swayam Prakash[202], Mostafa Qorbani[203], Amir Radfar[204,205], Anwar Rafay[206,207], Fakher Rahim[208,209], Robert C Reiner[48,86], Andre M. N. Renzaho[210], Leonardo Roever[211], Luca Ronfani[176], Basema Saddik[212], Roya Safari-Faramani[213], Sare Safi[214], Saeid Safiri[215], Payman Salamati[216,217], Yahya Salimi[186], Abdallah M. Samy[218], David C. Schwebel[219], Sadaf G. Sepanlou[147], Berrin Serdar[220,221], Masood Ali Shaikh[222], Mehran Shams-Beyranvand[223], Mahdi Sharif-Alhoseini[216], Jun She[224], Mika Shigematsu[225], Rahman Shiri[226], Reza Shirkoohi[227,228], Si Si[72], Dhirendra Narain Sinha[229], Jeffrey D Stanaway[48,86], Moslem Soofi[230], Mark A Stokes[231], Mu'awiyyah Babale Sufiyan[232], Rafael Tabarés-Seisdedos[233,234], Ken Takahashi[137], Segen Gebremeskel Tassew[67], Arash Tehrani-Banihashemi[181,235], Mohamad-Hani Temsah[236,153], Bach Xuan Tran[237,238], Khanh Bao Tran[239,240], Lorainne Tudor Car[241], Irfan Ullah[242,243], Pascual R

Valdez[244,245], Tommi Juhani Vasankari[246], Narayanaswamy Venketasubramanian[247,248], Francesco S Violante[249,250], Sergey Konstantinovitch Vladimirov[251,252], Vasily Vlassov[253], Giang Thu Vu[89], Gregory R Wagner[254], Yasir Waheed[255], Yuan-Pang Wang[256], Andrea Werdecker[257,258], Andrea Sylvia Winkler[259,260], Ebrahim M Yimer[67], Paul Yip[261,262], Biruck Desalegn Yirsaw[263], Engida Yisma[264], Naohiro Yonemoto[265], Mahmoud Yousefifard[266], Sojib Bin Zaman[267,268], Mohammad Zamani[269], Hamed Zandian[270,271], Yunquan Zhang[272,273], Sanjay Zodpey[274], Stephen S Lim[48,86].

Affiliations

1 Sydney School of Public Health, University of Sydney, Sydney, NSW, Australia.
2 Department of Epidemiology and Biostatistics, Imperial College London, London, UK.
3 School of Health Sciences, University of Manchester, Manchester, UK.
4 Section of Evidence Synthesis and Classification, International Agency for Research on Cancer, Lyon, France.
5 Rollins School of Public Health, Emory University, Atlanta, GA, USA.
6 Department of Medical Laboratory Sciences, Haramaya University, Harar, Ethiopia.
7 Department of Law, Philosophy, and Economic Studies, La Sapienza University, Rome, Italy.
8 Department of Preventive Medicine, Dongguk University, Gyeongju, South Korea.
9 Department of Community Medicine, Kathmandu University, Devdaha, Nepal.
10 College of Medicine, University College Hospital, Ibadan, Ibadan, Nigeria.
11 Department of Community Medicine, Zabol University of Medical Sciences, Zabol, Iran.
12 Department of Population Health Sciences, Duke University, Durham, NC, USA.
13 Duke Global Health Institute, Duke University, Durham, NC, USA.
14 Evidence Based Practice Center, Mayo Clinic Foundation for Medical Education and Research, Rochester, MN, USA.
15 Social Determinants of Health Research Center, Rafsanjan University of Medical Sciences, Rafsanjan, Iran.
16 Department of Health Management and Economics, Tehran University of Medical Sciences, Tehran, Iran.
17 Department of Health Policy and Administration, University of the Philippines Manila, Manila, Philippines.
18 Department of Applied Social Sciences, Hong Kong Polytechnic University, Hong Kong, China.
19 School of Health Sciences, Birmingham City University, Birmingham, UK.
20 The Judith Lumley Centre, La Trobe University, Melbourne, VIC, Australia.
21 General Office for Research and Technological Transfer, Peruvian National Institute of Health, Lima, Peru.
22 Faculty of Medicine, Alexandria University, Alexandria, Egypt.
23 School of Psychology, University of Auckland, Auckland, New Zealand.
24 Heidelberg Institute of Global Health (HIGH), Faculty of Medicine and University Hospital, Heidelberg University, Heidelberg, Germany.
25 T.H. Chan School of Public Health, Harvard University, Boston, MA, USA.
26 Department of Industrial Engineering, Pontifical Javeriana University, Bogota, Colombia.
27 Department of Community Medicine, Gandhi Medical College Bhopal, Bhopal, India.
28 Jazan University, Jazan, Saudi Arabia.
29 Social Determinants of Health Research Center, Lorestan University of Medical Sciences, Khorramabad, Iran.
30 Hepatitis Research Center, Lorestan University of Medical Sciences, Khorramabad, Iran.
31 Center for Research in Occupational Health, Pompeu Fabra University, Barcelona, Spain.
32 Emergency Hospital of Bucharest, Carol Davila University of Medicine and Pharmacy, Bucharest, Romania.
33 General Surgery Department, Carol Davila University of Medicine and Pharmacy, Bucharest, Romania.
34 Department of Statistical and Computational Genomics, National Institute of Biomedical Genomics, Kalyani, India.
35 Department of Statistics, University of Calcutta, Kolkata, India.
36 Social Determinants of Health Research Center, Babol University of Medical Sciences, Babol, Iran.

37 Department of Population and Health, Metropolitan Autonomous University, Mexico City, Mexico.
38 Department of Medical Epidemiology and Biostatistics, Karolinska Institutet, Stockholm, Sweden.
39 Applied Molecular Biosciences Unit, University of Porto, Porto, Portugal.
40 Institute of Public Health, University of Porto, Porto, Portugal.
41 Colombian National Health Observatory, National Institute of Health, Bogota, Colombia.
42 Epidemiology and Public Health Evaluation Group, National University of Colombia, Bogota, Colombia.
43 Mary MacKillop Institute for Health Research, Australian Catholic University, Melbourne, VIC, Australia.
44 School of Public Health, University of Hong Kong, Hong Kong, China.
45 Medical Research Council Lifecourse Epidemiology Unit, University of Southampton, Southampton, UK.
46 Department of Rheumatology, University of Oxford, Oxford, UK.
47 Public Health Foundation of India, Gurugram, India.
48 Institute for Health Metrics and Evaluation, University of Washington, Seattle, WA, USA.
49 Institute for Global Health Innovations, Duy Tan University, Hanoi, Vietnam.
50 Toxoplasmosis Research Center, Mazandaran University of Medical Sciences, Sari, Iran.
51 School of Nutrition, Food Science and Technology, Hawassa University, Hawassa, Ethiopia.
52 Department of Community Medicine, University of Peradeniya, Peradeniya, Sri Lanka.
53 Faculty of Medicine, University of Belgrade, Belgrade, Serbia.
54 Department of Public Health Sciences, Karolinska Institutet, Stockholm, Sweden.
55 Multiple Sclerosis Research Center, Tehran University of Medical Sciences, Tehran, Iran.
56 College of Medicine, Imam Muhammad Ibn Saud Islamic University, Riyadh, Saudi Arabia.
57 Department of Psychology, Federal University of Sergipe, Sao Cristovao, Brazil.
58 Department of Neurobiology, Care Sciences and Society, Karolinska Institutet, Stockholm, Sweden.
59 Division of Neurology, University of Ottawa, Ottawa, ON, Canada.
60 REQUIMTE/LAQV, University of Porto, Porto, Portugal.
61 Department of Psychiatry, Kaiser Permanente, Fontana, CA, USA.
62 Department of Health Sciences, A.T. Still University, Mesa, AZ, USA.
63 Department of Public Health Medicine, Bielefeld University, Bielefeld, Germany.
64 Gene Expression & Regulation Program, Cancer Institute (W.I.A.), Philadelphia, PA, USA.
65 Department of Dermatology, Kobe University, Kobe, Japan.
66 Department of Environmental Health Science, Mario Negri Institute for Pharmacological Research, Milan, Italy.
67 School of Pharmacy, Mekelle University, Mekelle, Ethiopia.
68 Department of Biostatistics, Mekelle University, Mekelle, Ethiopia.
69 Adelaide Medical School, University of Adelaide, Adelaide, SA, Australia.
70 Graduate Studies in Epidemiology, Universidade Federal do Rio Grande do Sul, Porto Alegre, Brazil.
71 School of Medicine, Boston University, Boston, MA, USA.
72 School of Public Health and Preventive Medicine, Monash University, Melbourne, VIC, Australia.
73 Department of Epidemiology and Biostatistics, College of Public Health, Zhengzhou University, Zhengzhou, China.
74 March of Dimes, Arlington, VA, USA.
75 School of Public Health, West Virginia University, Morgantown, WV, USA.
76 Department of Pharmacology, Tehran University of Medical Sciences, Tehran, Iran.
77 Obesity Research Center, Research Institute for Endocrine Sciences, Shahid Beheshti University of Medical Sciences, Tehran, Iran.

78 Department of Radiology, Johns Hopkins University, Baltimore, MD, USA.
79 Department of Family and Community Medicine, Arabian Gulf University, Manama, Bahrain.
80 School of Health and Environmental Studies, Hamdan Bin Mohammed Smart University, Dubai, United Arab Emirates.
81 Department of Health Education & Promotion, Kermanshah University of Medical Sciences, Kermanshah, Iran.
82 School of Nursing and Midwifery, Tabriz University of Medical Sciences, Tabriz, Iran.
83 Independent Consultant, Tabriz, Iran.
84 Department of Public Health, University of Massachusetts Lowell, Lowell, MA, USA.
85 Occupational Health Surveillance Program, Massachusetts Department of Public Health, Boston, MA, USA.
86 Department of Health Metrics Sciences, School of Medicine, University of Washington, Seattle, WA, USA.
87 Department of Neurology, Cairo University, Cairo, Egypt.
88 Department of Public Health, Mizan-Tepi University, Teppi, Ethiopia.
89 Center of Excellence in Behavioral Medicine, Nguyen Tat Thanh University, Ho Chi Minh, Vietnam.
90 Department of Pediatrics, Dell Medical School, University of Texas Austin, Austin, TX, USA.
91 Social Determinants of Health Research Center, Guilan University of Medical Sciences, Rasht, Iran.
92 Guilan Road Trauma Research Center, Guilan University of Medical Sciences, Rasht, Iran.
93 Department of Pharmacology and Therapeutics, University of Dhaka, Dhaka, Bangladesh.
94 Health System and Population Studies Division, International Centre for Diarrhoeal Disease Research Bangladesh, Dhaka, Bangladesh.
95 Department of Epidemiology and Biostatistics, Tehran University of Medical Sciences, Tehran, Iran.
96 Faculty of Dentistry, Department of Legal Medicine and Bioethics, Carol Davila University of Medicine and Pharmacy, Bucharest, Romania.
97 Clinical Legal Medicine, National Institute of Legal Medicine Mina Minovici, Bucharest, Romania.
98 Department of Epidemiology and Health Statistics, Central South University, Changsha, China.
99 Department of Community Medicine, College of Medicine, University of Ibadan, Ibadan, Nigeria.
100 Research Institute for Endocrine Sciences, Shahid Beheshti University of Medical Sciences, Tehran, Iran.
101 Non-communicable Diseases Research Center, Tehran University of Medical Sciences, Tehran, Iran.
102 Institute for Physical Activity and Nutrition, Deakin University, Burwood, VIC, Australia.
103 Sydney Medical School, University of Sydney, Sydney, NSW, Australia.
104 Department of Global Health, Economics and Policy, Faculty of Medical Sciences, University of Kragujevac, Kragujevac, Serbia.
105 Division of Health Economics, Lund University, Lund, Sweden.
106 Department of Community Medicine, Banaras Hindu University, Varanasi, India.
107 Department of Ophthalmology, Heidelberg University, Mannheim, Germany.
108 Beijing Institute of Ophthalmology, Beijing Tongren Hospital, Beijing, China.
109 Social Determinants of Health Research Center, University of Social Welfare and Rehabilitation Sciences, Tehran, Iran.
110 Department of Family Medicine and Public Health, University of Opole, Opole, Poland.
111 Faculty of Medicine and Health Sciences, University of Opole, Opole, Poland.
112 Institute of Family Medicine and Public Health, University of Tartu, Tartu, Estonia.
113 Department of Nutrition and Dietetics, Mekelle University, Mekelle, Ethiopia.
114 Department of Epidemiology, Hamadan University of Medical Sciences, Hamadan, Iran.

115 Department of Epidemiology, School of Public Health and Safety, Shahid Beheshti University of Medical Sciences, Tehran, Iran.
116 Immunogenetics Research Center, Mazandaran University of Medical Sciences, Sari, Iran.
117 Department of Neurology, Mazandaran University of Medical Sciences, Sari, Iran.
118 Hematology-Oncology and Stem Cell Transplantation Research Center, Tehran University of Medical Sciences, Tehran, Iran.
119 Hematologic Malignancies Research Center, Tehran University of Medical Sciences, Tehran, Iran.
120 Department of Mental Health, University of Tokyo, Tokyo, Japan.
121 Department of Public Health and Community Medicine, Jordan University of Science and Technology, Ramtha, Jordan.
122 Epidemiology and Biostatistics Department, Health Services Academy, Islamabad, Pakistan.
123 Department of Nutrition and Health Science, Ball State University, Muncie, IN, USA.
124 School of Medicine, Xiamen University Malaysia, Sepang, Malaysia.
125 Department of Health Management and Health Economics, Kristiania University College, Oslo, Norway.
126 Department of Health Services Policy and Management, University of South Carolina, Columbia, SC, USA.
127 Independent Consultant, Jakarta, Indonesia.
128 Department of Internal and Pulmonary Medicine, Sheri Kashmir Institute of Medical Sciences, Srinagar, India.
129 CIBERSAM, San Juan de Dios Sanitary Park, Sant Boi de Llobregat, Spain.
130 Catalan Institution for Research and Advanced Studies (ICREA), Barcelona, Spain.
131 Department of Demography, University of Montreal, Montreal, QC, Canada.
132 Department of Social and Preventive Medicine, University of Montreal, Montreal, QC, Canada.
133 Department of Psychiatry, University of Nairobi, Nairobi, Kenya.
134 Department of Psychology, University College London, London, UK.
135 Department of Community and Family Medicine, Academy of Medical Science, Baghdad, Iraq.
136 Department of Public Health, Maragheh University of Medical Sciences, Maragheh, Iran.
137 Asbestos Diseases Research Institute, University of Sydney, Sydney, NSW, Australia.
138 Department of Prevention, Local Health Unit Tuscany Centre, Florence, Italy.
139 Department of Health Sciences, University of Florence, Florence, Italy.
140 School of Public Health, University of Haifa, Haifa, Israel.
141 Department of Epidemiology and Biostatistics, University of the Philippines Manila, Manila, Philippines.
142 World Surgical Foundation Philippines, Manila, Philippines.
143 General Surgery Department, Aintree University Hospital National Health Service (NHS) Foundation Trust, Liverpool, UK.
144 Surgery Department, University of Liverpool, Liverpool, UK.
145 Institute of Medicine, Tribhuvan University, Kathmandu, Nepal.
146 Department of Public Health, Trnava University, Trnava, Slovakia.
147 Digestive Diseases Research Institute, Tehran University of Medical Sciences, Tehran, Iran.
148 Non-Communicable Diseases Research Center, Shiraz University of Medical Sciences, Shiraz, Iran.
149 Division of Plastic Surgery, University of Washington, Seattle, WA, USA.
150 Department of Internal Medicine, SevenHills Hospital, Mumbai, India.
151 Department of Medical Laboratory Science, Bahir Dar University, Bahir Dar, Ethiopia.
152 Research Department Prince Mohammed Bin Abdulaziz Hospital, Ministry of Health, Riyadh, Saudi Arabia.
153 College of Medicine, Alfaisal University, Riyadh, Saudi Arabia.
154 Peru Country Office, United Nations Population Fund (UNFPA), Lima, Peru.
155 School of Pharmacy, Haramaya University, Harar, Ethiopia.

156 Department of Pharmacy, Wollo University, Dessie, Ethiopia.
157 Neurocenter, Helsinki University Hospital, Helsinki, Finland.
158 School of Health Sciences, University of Melbourne, Parkville, VIC, Australia.
159 Breast Surgery Unit, Helsinki University Hospital, Helsinki, Finland.
160 Clinical Microbiology and Parasitology Unit, Dr. Zora Profozic Polyclinic, Zagreb, Croatia.
161 University Centre Varazdin, University North, Varazdin, Croatia.
162 Center for Innovation in Medical Education, Pomeranian Medical University, Szczecin, Poland.
163 Department of Hypertension, Pomeranian Medical University, Szczecin, Poland.
164 Pacific Institute for Research & Evaluation, Calverton, MD, USA.
165 School of Public Health, Curtin University, Perth, WA, Australia.
166 Global Institute of Public Health, Ananthapuri Hospitals and Research Institute, Trivandrum, India, Trivandrum, India.
167 Achutha Menon Centre for Health Science Studies, Sree Chitra Tirunal Institute for Medical Sciences and Technology, Trivandrum, India.
168 Faculty of General Medicine, Kyrgyz State Medical Academy, Bishkek, Kyrgyzstan.
169 Department of Atherosclerosis and Coronary Heart Disease, National Center of Cardiology and Internal Disease, Bishkek, Kyrgyzstan.
170 Institute of Public Health, Heidelberg University, Heidelberg, Germany.
171 Institute of Addiction Research (ISFF), Frankfurt University of Applied Sciences, Frankfurt, Germany.
172 Department of Biostatistics, Hamadan University of Medical Sciences, Hamadan, Iran.
173 Health Systems and Policy Research Unit, Ahmadu Bello University, Zaria, Nigeria.
174 Iran National Institute of Health Research, Tehran University of Medical Sciences, Tehran, Iran.
175 Faculty of Life Sciences and Medicine, King's College London, London, UK.
176 Clinical Epidemiology and Public Health Research Unit, Burlo Garofolo Institute for Maternal and Child Health, Trieste, Italy.
177 Department of Public Health Medicine, University of KwaZulu-Natal, Durban, South Africa.
178 Health Sciences Research Center, Mazandaran University of Medical Sciences, Sari, Iran.
179 Social Determinants of Health Research Center, Kurdistan University of Medical Sciences, Sanandaj, Iran.
180 Department of Epidemiology and Biostatistics, Kurdistan University of Medical Sciences, Sanandaj, Iran.
181 Preventive Medicine and Public Health Research Center, Iran University of Medical Sciences, Tehran, Iran.
182 International Laboratory for Air Quality and Health, Queensland University of Technology, Brisbane, QLD, Australia.
183 Department of Surgery, University of Washington, Seattle, WA, USA.
184 Department of Pediatric Medicine, Nishtar Medical University, Multan, Pakistan.
185 Department of Pediatrics, Institute of Mother & Child Care, Multan, Pakistan.
186 Department of Epidemiology & Biostatistics, Kermanshah University of Medical Sciences, Kermanshah, Iran.
187 Suraj Eye Institute, Nagpur, India.
188 Anatomy and Embryology Department, Carol Davila University of Medicine and Pharmacy, Bucharest, Romania.
189 Department of Cardiology, Cardio-Aid, Bucharest, Romania.
190 Faculty of Health Sciences, University of Tampere, Tampere, Finland.
191 Centre of Cardiovascular Research and Education in Therapeutics, Monash University, Melbourne, VIC, Australia.
192 Independent Consultant, Accra, Ghana.
193 Translational Health Research Institute, Western Sydney University, Penrith, NSW, Australia.

194 Department of Psychiatry and Behavioural Neurosciences, McMaster University, Hamilton, ON, Canada.
195 Department of Psychiatry, University of Lagos, Lagos, Nigeria.
196 Centre for Healthy Start Initiative, Lagos, Nigeria.
197 Analytical Center, Moscow Institute of Physics and Technology, Dolgoprudny, Russia.
198 Committee for the Comprehensive Assessment of Medical Devices and Information Technology, Health Technology Assessment Association, Moscow, Russia.
199 Department of TB & Respiratory Medicine, Jagadguru Sri Shivarathreeswara University, Mysore, India.
200 Augenpraxis Jonas, Heidelberg University, Heidelberg, Germany.
201 Department of Medical Humanities and Social Medicine, Kosin University, Busan, South Korea.
202 Department of Nephrology, Sanjay Gandhi Postgraduate Institute of Medical Sciences, Lucknow, India.
203 Non-communicable Diseases Research Center, Alborz University of Medical Sciences, Karaj, Iran.
204 Journal of Environmental and Public Health, Hindawi, London, UK.
205 MEDICHEM, Barcelona, Spain.
206 Department of Epidemiology & Biostatistics, Contech School of Public Health, Lahore, Pakistan.
207 Contech International Health Consultants, Lahore, Pakistan.
208 Thalassemia and Hemoglobinopathy Research Center, Ahvaz Jundishapur University of Medical Sciences, Ahvaz, Iran.
209 Clinical Research Development Unit, Golestan Hospital, Ahvaz Jundishapur University of Medical Sciences, Ahvaz, Iran.
210 School of Social Sciences and Psychology, Western Sydney University, Penrith, NSW, Australia.
211 Department of Clinical Research, Universidade Federal de Uberlândia, Uberlândia, Brazil.
212 Medical Department, University of Sharjah, Sharjah, United Arab Emirates.
213 Faculty of Public Health, Kermanshah University of Medical Sciences, Kermanshah, Iran.
214 Ophthalmic Epidemiology Research Center, Shahid Beheshti University of Medical Sciences, Tehran, Iran.
215 Department of Epidemiology and Biostatistics, School of Public Health, Tabriz University of Medical Sciences, Tabriz, Iran.
216 Sina Trauma and Surgery Research Center, Tehran University of Medical Sciences, Tehran, Iran.
217 School of Health and Policy Management, Faculty of Health, York University, Toronto, ON, Canada.
218 Department of Entomology, Ain Shams University, Cairo, Egypt.
219 Department of Psychology, University of Alabama at Birmingham, Birmingham, AL, USA.
220 Environmental Health Associates LLC, Englewood, CO, USA.
221 School of Public Health, University of Colorado Denver, Denver, CO, USA.
222 Independent Consultant, Karachi, Pakistan.
223 School of Medicine, Dezful University of Medical Sciences, Dezful, Iran.
224 Department of Pulmonary Medicine, Fudan University, Shanghai, China.
225 National Institute of Infectious Diseases, Tokyo, Japan.
226 Finnish Institute of Occupational Health, Helsinki, Finland.
227 Cancer Research Institute, Tehran University of Medical Sciences, Tehran, Iran.
228 Cancer Biology Research Center, Tehran University of Medical Sciences, Tehran, Iran.
229 Department of Epidemiology, School of Preventive Oncology, Patna, India.
230 Social Development and Health Promotion Research Center, Kermanshah University of Medical Sciences, Kermanshah, Iran.

231 Department of Psychology, Deakin University, Burwood, VIC, Australia.
232 Department of Community Medicine, Ahmadu Bello University, Zaria, Nigeria.
233 Department of Medicine, University of Valencia, Valencia, Spain.
234 Carlos III Health Institute, Biomedical Research Networking Center for Mental Health Network (CiberSAM), MADRID, Spain.
235 Department of Community Medicine, Iran University of Medical Sciences, Tehran, Iran.
236 Department of Pediatrics, King Saud University, Riyadh, Saudi Arabia.
237 Department of Health Economics, Hanoi Medical University, Hanoi, Vietnam.
238 Bloomberg School of Public Health, Johns Hopkins University, Baltimore, MD, USA.
239 Department of Molecular Medicine and Pathology & Auckland Cancer Society Research Centre, University of Auckland, Auckland, New Zealand.
240 Maurice Wilkins Centre for Biodiscovery, Auckland, New Zealand.
241 Lee Kong Chian School of Medicine, Nanyang Technological University, Singapore, Singapore.
242 Gomal Center of Biochemistry and Biotechnology, Gomal University, Dera Ismail Khan, Pakistan.
243 TB Culture Laboratory, Mufti Mehmood Memorial Teaching Hospital, Dera Ismail Khan, Pakistan.
244 Argentine Society of Medicine, Buenos Aires, Argentina.
245 Hospital Velez Sarsfield, Buenos Aires, Argentina.
246 UKK Institute, Tampere, Finland.
247 Raffles Neuroscience Centre, Raffles Hospital, Singapore, Singapore.
248 Yong Loo Lin School of Medicine, National University of Singapore, Singapore, Singapore.
249 Department of Medical and Surgical Sciences, University of Bologna, Bologna, Italy.
250 Occupational Health Unit, Sant'Orsola Malpighi Hospital, Bologna, Italy.
251 Moscow Institute of Physics and Technology, Moscow, Russia.
252 Department of Information and Internet Technologies, I.M. Sechenov First Moscow State Medical University, Moscow, Russia.
253 Department of Health Care Administration and Economy, National Research University Higher School of Economics, Moscow, Russia.
254 Department of Environmental Health, Harvard University, Boston, MA, USA.
255 Foundation University Medical College, Foundation University Islamabad, Rawalpindi, Pakistan.
256 Department of Psychiatry, University of São Paulo, São Paulo, Brazil.
257 Demographic Change and Ageing Research Area, Federal Institute for Population Research, Wiesbaden, Germany.
258 Independent Consultant, Staufenberg, Germany.
259 Institute of Health and Society, University of Oslo, Oslo, Norway.
260 Department of Neurology, Technical University of Munich, Munich, Germany.
261 Centre for Suicide Research and Prevention, University of Hong Kong, Hong Kong, China.
262 Department of Social Work and Social Administration, University of Hong Kong, Hong Kong, China.
263 University of South Australia, Adelaide, NSW, Australia.
264 School of Allied Health Sciences, Addis Ababa University, Addis Ababa, Ethiopia.
265 Department of Psychopharmacology, National Center of Neurology and Psychiatry, Tokyo, Japan.
266 Physiology Research Center, Iran University of Medical Sciences, Tehran, Iran.
267 Maternal and Child Health Division, International Centre for Diarrhoeal Disease Research, Bangladesh, Dhaka, Bangladesh.
268 Department of Medicine, School of Clinical Sciences at Monash Health, Monash University, Melbourne, VIC, Australia.
269 Student Research Committee, Babol University of Medical Sciences, Babol, Iran.

270 Social Determinants of Health Research Center, Ardabil University of Medical Science, Ardabil, Iran.
271 Department of Community Medicine, Ardabil University of Medical Sciences, Ardabil, Iran.
272 School of Public Health, Wuhan University of Science and Technology, Wuhan, China.
273 Department of Preventative Medicine, Wuhan University, Wuhan, China.
274 Indian Institute of Public Health, Public Health Foundation of India, Gurugram, India.
